# Rejuvenation of Senescent Bone Marrow Mesenchymal Stromal Cells by Pulsed Triboelectric Stimulation

**DOI:** 10.1002/advs.202100964

**Published:** 2021-07-14

**Authors:** Gaocai Li, Qianqian Zhu, Bingjin Wang, Rongjin Luo, Xiaohui Xiao, Yi Zhang, Liang Ma, Xiaobo Feng, Jingang Huang, Xuhui Sun, Zhen Wen, Yue Pan, Cao Yang

**Affiliations:** ^1^ Department of Orthopaedics Union Hospital Tongji Medical College Huazhong University of Science and Technology Wuhan 430022 China; ^2^ Institute of Functional Nano and Soft Materials (FUNSOM) Jiangsu Key Laboratory for Carbon‐Based Functional Materials and Devices Soochow University Suzhou 215123 China; ^3^ Guangdong Provincial Key Laboratory of Malignant Tumor Epigenetics and Gene Regulation Guangdong‐Hong Kong Joint Laboratory for RNA Medicine Department of Cardiology Medical Research Center Sun Yat‐Sen Memorial Hospital Sun Yat‐Sen University Guangzhou 510120 P. R. China

**Keywords:** electrical stimulation, mesenchymal stromal cells, osteogenesis, pulsed current, rejuvenation, triboelectric nanogenerator

## Abstract

Stem cell senescence contributes to stem cell exhaustion and drives various aging‐associated disorders. However, strategies to rejuvenate senescent stem cells are limited. The present study proposes an approach based on triboelectric stimulation to rejuvenate senescent bone marrow mesenchymal stromal cells (BMSCs) by fabricating a pulsed triboelectric nanogenerator (P‐TENG) that can produce stable pulsed current output unaffected by the triggered frequency. The senescence phenotypes of aged BMSCs are reversed by triboelectric stimulation at 30 µA at 1.5 Hz. Triboelectric stimulation enhances the proliferation of aged BMSCs and increases their pluripotency and differentiation capacity. Additionally, mechanistic investigations reveal that pulsed triboelectric stimulation by P‐TENG rejuvenates senescent BMSCs by enhancing MDM2‐dependent p53 degradation, which is demonstrated by loss‐of‐function studies of MDM2 and p53. Overall, this study identifies a new approach for the rejuvenation of senescent BMSCs and describes a promising therapeutic intervention for many diseases associated with aged BMSCs.

## Graphical abstract

A pulsed triboelectric nanogenerator system is fabricated to investigate the effect of triboelectric stimulation on senescent bone marrow mesenchymal stromal cells (BMSCs) from aged donors. This electrical stimulation can promote the cellular proliferation and increase the pluripotency of senescent BMSCs, and promote the chondrogenesis and osteogenesis meanwhile, indicating the pro‐youth effect and a potential rejuvenation tactic to counter senescence of aged BMSCs.

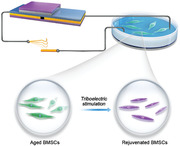

## 1 Introduction

Aging is a complex physiological process that can lead to the degeneration and dysfunction of multiple tissues and organs.^[^ ^1^ ^]^ Stem cells are prone to senescence during organismal aging owing to profound age‐related changes in stem cell niches and intracellular biological dysfunctions.^[^ ^2^ ^]^ Recently, stem cell aging has been recognized as an important negative factor in organismal aging and a critical driver of various aging‐associated degenerative diseases.^[^ ^3^ ^]^ Notably, aged mesenchymal stromal cells (MSCs) have limited proliferation and differentiation potential, which leads to senescence and results in osteoporosis and osteoarthritis.^[^ ^4^ ^]^ Therefore, it is important to maintain stemness and repair capacity of stem cells. Several rejuvenating strategies have been developed to reverse senescence of stem cells by targeting the relevant intrinsic cell signaling pathways, intervening in epigenetic modifications, reversing DNA damage, and clearing senescent stem cells.^[^ ^3^, ^5^ ^]^ However, these strategies have not been applied extensively owing to their limited efficiency and formidable limitations; thus, it is important to explore new and efficient tactics to rejuvenate senescent stem cells.

Electrical fields and electrical signaling are essential for many cellular processes because they are fundamental for vital activities.^[^ ^6^ ^]^ Several studies indicated that triboelectric stimulation produced by triboelectric nanogenerator (TENG) including direct current or electric field can influence cellular proliferation and regeneration.^[^ ^7^ ^]^ Based on the coupling effect of triboelectrification and electrostatic induction,^[^ ^8^ ^]^ the effect of triboelectric stimulation on biological activities and cellular processes has been delved in depth. Recent studies have focused on the effects of TENGs on biological activities and cellular processes. TENGs can induce reprogramming and proliferation of fibroblasts for wound skin healing,^[^ ^9^ ^]^ and also stimulate the proliferation and differentiation of osteoblasts and provide a therapy for osteoporosis.^[^ ^10^ ^]^ Additionally, triboelectric stimulation can enhance the proliferation and neural differentiation of MSCs.^[^ ^11^ ^]^ Furthermore, wound healing and hair regeneration can be promoted by consistent voltage pulse stimulation from a TENG‐based self‐powered system.^[^ ^12^ ^]^ These studies revealed potential applications of triboelectric stimulation in biomedical practice.

MSCs are multipotent cells that can differentiate into multiple tissues of the mesoderm. MSCs can be isolated from many tissues, including bone marrow, muscle, skin, intestine, adipose tissue, umbilical cord, renal, and epithelium. Bone marrow‐derived mesenchymal stromal cells (BMSCs) are representative stem cells extensively used in tissue engineering and regenerative medicine because they are readily available and are easy to isolate and culture. However, BMSCs from aged individuals showed decreased function and have limited use for regeneration.^[^ ^13^ ^]^ Comparison of BMSCs from aged donors with those from young donors demonstrated a significant decline in the pluripotency and proliferation capacity of BMSCs from aged donors, which indicates senescence of aged BMSCs. Several studies demonstrated that stemness and proliferation capacity of stem cells can be regulated and improved,^[^ ^14^ ^]^ there remains a huge demand to explore more effective tactics. Inspired by recent advances of triboelectric technology in biomedical applications and therapeutic strategies,^[^ ^9^, ^15^ ^]^ we explored whether the triboelectric stimulation can be used as a rejuvenating intervention in senescent BMSCs.

This study reports that triboelectric stimulation can rejuvenate the BMSCs of aged human donors using a pulsed TENG (P‐TENG) that produces stable pulsed current output unaffected by triggered frequency.^[^ ^16^ ^]^ Comparison with untreated BMSCs indicated that BMSCs triboelectrically stimulated with a pulsed current of 30 µA at 1.5 Hz manifested alleviated senescent phenotypes and increased proliferation and pluripotency, which were also demonstrated in in vivo experiments. Furthermore, the results demonstrated an essential role of the MDM2‐p53 pathway in rejuvenation, and inhibition of MDM2 reduced the rejuvenating effect of triboelectric stimulation on senescent BMSCs. Thus, our study is the first to reveal that aged BMSCs can be rejuvenated by triboelectric stimulation and provides a novel approach for rejuvenation of senescent MSCs for aging patients, emphasizing a promising therapeutic intervention for the treatment of osteoporosis, osteoarthritis, and other aging‐related diseases.

## 2 Results and Discussion

**Figure 1:**
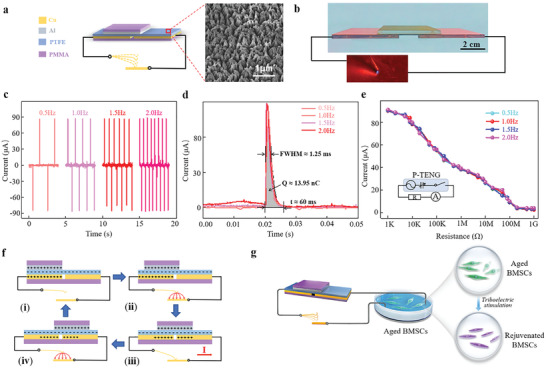
Characterizations and electrical performance of pulsed triboelectric nanogenerator (P‐TENG). a) Schematic illustration of a P‐TENG with the inset showing the top‐view SEM image of PTFE. b) Image of a fabricated P‐TENG and the spark generated by the potential difference. c) Short‐circuit current (*I*_sc_) at vibration frequencies of 0.5, 1.0, 1.5, and 2.0 Hz. d) Magnified current peaks at various vibration frequencies. e) Output current under various resistances at various vibration frequencies. Inset shows the equivalent circuit diagram. f) A cycle of the electricity generation process illustrating the working mechanism of P‐TENG. g) Schematic illustration of rejuvenation of aged BMSCs by triboelectric stimulation with P‐TENG.

**Figure****1**a shows the schematic structure of a P‐TENG with an electrostatic vibrator switch. The action of P‐TENG is based on the sliding mode. A polymethyl methacrylate (PMMA) sheet with a thickness of 3 mm was used as the substrate to fabricate a P‐TENG. A square Al foil with a side length of 5 cm and a thickness of 25 µm was used as a triboelectric layer. Two pieces of Cu foil with a thickness of 50 µm were used as two electrodes of P‐TENG. A polytetrafluoroethylene (PTFE) film was attached to Cu foil as another triboelectric layer. The top‐view scanning electron microscopy (SEM) image of the PTFE film after triboelectrification shows nanowires ≈100 nm in diameter and ≈500 nm in length (Figure 1a). Figure 1b presents an image of a fabricated P‐TENG. A spark is generated by the potential difference when the switch is closed (Video S1, Supporting Information). The short‐circuit current (*I*_sc_) of the P‐TENG is stable at ≈88 µA at vibration frequencies of 0.5, 1.0, 1.5, and 2.0 Hz (Figure 1c). The magnifications of a single current peak of the P‐TENG at four various vibration frequencies are illustrated in Figure 1d. The current increases directly from zero to the peak and then slowly decreases from the peak to zero within ≈60 ms. The full width at half maximum (FWHM) of the four peaks is ≈1.25 ms, and the integral area of each peak is ≈13.95 nC. In Figure 1e, the resistance load output current decreases from ≈88 to ≈3 µA as the load resistance increases from 1 kΩ to 1 GΩ, and the variation trends are essentially similar at various frequencies. These electrical properties are due to specific structure of P‐TENG.^[^ ^17^ ^]^ Figure 1f shows the working mechanism of P‐TENG. In the initial state (i), the Al foil overlaps with a part of the left Cu electrode, and the PTFE film and the left Cu electrode produce negative and positive charges, respectively, to achieve electrostatic equilibrium. At this state, there is no potential difference between the two Cu electrodes. When the triboelectric layer slides rightward (state ii), the negative charges are separated from the positive charges on the left Cu electrode to generate a difference in the potentials between the two Cu electrodes. An electrostatic attraction force between the vibrator and the Cu plate is thus generated to drive the approach of the vibrator to the Cu plate. The vibrator does not physically contact the Cu plate, which means that the switch is open. Therefore, no current is generated between the two Cu electrodes. Sliding of Al foil to state iii connects the two Cu electrodes to close the switch; thus, the positive charges flow from the left electrode to the right electrode. This process results in the generation of a transient pulse current between the two Cu electrodes. Subsequently, the electrostatic balance is reached, and the electrostatic attraction disappears, reducing the difference of the potentials between the two Cu electrodes to zero. The Al foil continues to slide left (state iv). These states complete the half‐cycle of the current generation process. Then, the Al foil slides from the right to the left and generates the opposite current thus repeating these processes and periodically generating the pulse current. Furthermore, to explore the biofunctional effect of triboelectric stimulation on senescent BMSCs, a stimulation system including a P‐TENG was designed and connected to a cell culture dish in which BMSCs was cultured (Figure 1g).

**Figure 2:**
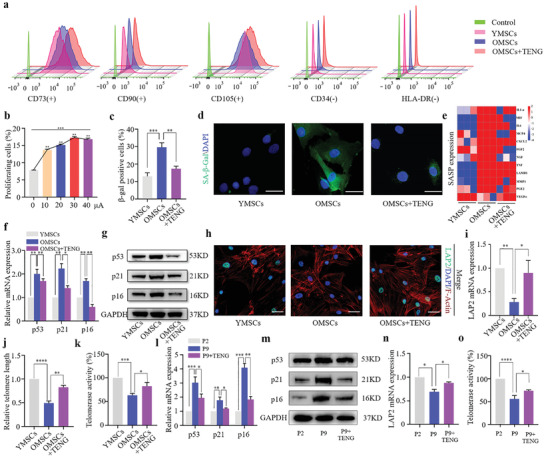
Senescence of aged BMSCs prevented by P‐TENG. a) FACS analysis of BMSC surface markers CD73, CD90, CD105, CD34, and HLA‐DR. b) EdU labeling and FACS analysis of cellular proliferation stimulated at various currents (0, 10, 20, 30, and 40 µA, *n* = 3). Statistical evaluation was performed by ANOVA; the data are shown as the mean ± SD. c) The percentage of *β*‐galactosidase‐positive cells is shown as the mean ± SD (*n* = 3). d) Fluorescence staining for *β*‐galactosidase (green) using the C_12_FDG substrate; nuclei stained DAPI (blue; scale bar: 20 µm). e) The expression level of SASP shown as a heat map using RT‐qPCR (*n* = 3). f,g) The protein and mRNA levels of p53, p21, and p16 determined using western blot and RT‐qPCR (*n* = 3). Expression levels were normalized to GAPDH, and the results are presented as the mean ± SD. h) Immunofluorescence staining of LAP2 (green), nucleus (blue), and F‐actin (red) (scale bar: 20 µm). i) Expression of LAP2 assayed by RT‐qPCR (*n* = 3). j) Relative telomere length measured by RT‐qPCR (*n* = 3). k) Relative telomerase activity shown of YMSCs, OMSCs, and TENG stimulated OMSCs (*n* = 3). l,m) The protein and mRNA levels of p53, p21, and p16 in passage 2 (P2), passage 9 YMSCs (P9), and TENG‐stimulated P9, determined using western blot and RT‐qPCR (*n* = 3). Expression levels were normalized to GAPDH, and the results are presented as the mean ± SD (*n* = 3). n) Expression of LAP2 assayed by RT‐qPCR (*n* = 3). o) Relative telomerase activity shown of different passage YMSCs with stimulation or not (*n* = 3). Statistical evaluation was performed by one‐way ANOVA with Dunnett's multiple comparison test (*p < 0.05, **p < 0.01, ***p < 0.001).

Stem cell senescence accounts for most of the decline in homeostatic maintenance and tissue regenerative capacity associated with advanced aging. It is not known whether triboelectric stimulation can be used to counteract the cellular senescence of aged BMSCs. We tested the influence of triboelectric stimulation on senescent BMSCs using P‐TENG to stimulate aged BMSCs for 7 d with 30 min of stimulation per day. Control groups of aged and normal BMSCs were cultured under the same conditions except for electrical stimulation. FACS analysis indicated that all senescent BMSCs (OMSCs stimulated with P‐TENG and control) and normal BMSCs (YMSCs) were positive for typical BMSC markers, including CD73, CD90, and CD105, and negative for BMSC‐irrelevant antigens, such as CD34 and HLA‐DR. This result indicated that electrical stimulation did not affect the identity of BMSCs (**Figure** **2**a). EdU labeling combined with FACS analysis was used to analyze cellular proliferation stimulated by various electrical currents. The current of 30 µA had maximum antisenescence effect, and the stimulatory effect was decreased if the current was higher than 30 µA (Figure 2b and Figure S1a, Supporting Information). Senescence‐associated *β*‐galactosidase staining indicated that senescent BMSCs from elderly donors were more senescent than normal BMSCs from young donors. Exposure of senescent BMSCs to TENG decreased senescence‐associated *β*‐galactosidase activity compared with that in control senescent BMSCs (Figure 2c and Figure S1b, Supporting Information). Additionally, *β*‐galactosidase fluorescence staining of BMSCs using C_12_FDG substrate confirmed these results (Figure 2d). Analysis of the senescence‐associated secretory phenotypes (SASP) by quantitative reverse transcription polymerase chain reaction (RT‐qPCR) showed that stimulation of senescent BMSCs by P‐TENG decreased the expression of SASP (Figure 2e). Senescence markers of BMSCs, p53, p21, and p16 were assayed using western blot and RT‐qPCR. The results revealed that triboelectric stimulation inhibited the expression of aging‐associated biomarkers and alleviated the senescence of aged BMSCs (Figure 2f,g). The level of LAP2 was assayed by immunofluorescence analysis and RT‐qPCR to evaluate the effect of pulsed electrical stimulation (Figure 2h,i and Figure S1c, Supporting Information). Additionally, stimulated OMSCs showed markedly prolonged telomere lengths and higher telomerase activity compared with the control group (Figure 2j,k). Meanwhile, the replicative senescence of passage 9 (p9) YMSCs could be abolished by triboelectric stimulation to some extent, indicated by downregulated senescence markers and increased telomerase activity (Figure 2l–n). The data indicated that triboelectric stimulation could inhibit the senescence of BMSCs.

**Figure 3:**
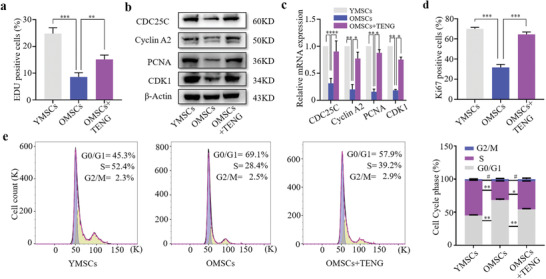
Proliferation of senescent BMSCs enhanced by triboelectric stimulation. a) Percentage of EdU‐positive cells shown as the mean ± SD (*n* = 3). b,c) Expression of proliferation‐associated markers analyzed using western blot and RT‐qPCR (*n* = 3). Expression of *β*‐actin was used as a control. d) Ki67‐positive cells (*n* = 3). e) Cell cycle analysis performed by FACS (*n* = 3). Statistical evaluation was performed by one‐way ANOVA with Dunnett's multiple comparison test (^#^ no significance, *p < 0.05, **p < 0.01, ***p < 0.001).

EdU incorporation analysis showed that the percentage of proliferating cells was higher after P‐TENG treatment (**Figure** **3**a and Figure S2a, Supporting Information). This result demonstrated that electrical stimulation improved the proliferation of senescent BMSCs. The cell proliferation markers, including CDC25C, cyclin‐A2, PCNA, and CDK1, were assayed using western blot and RT‐qPCR to evaluate the stimulation of the proliferation of BMSCs. Electrical pulse stimulation increased the expression of these markers, indicating enhanced proliferation of senescent BMSCs (Figure 3b,c). Moreover, immunofluorescence analysis of Ki67 showed higher expression of Ki67 in TENG‐stimulated senescent BMSCs (Figure 3d and Figure S2b, Supporting Information). Cell cycle analysis showed that triboelectric stimulation rescued the senescence of aged BMSCs. ≈39.2% of the cells were in the synthesis (S) phase after electrical treatment, and only 28.4% of the cells were in the S phase in the control group. This result demonstrated that higher proportion of BMSCs underwent cell division and DNA replication (Figure 3e) and confirmed that triboelectric stimulation enhanced the proliferation of senescent BMSCs.

**Figure 4:**
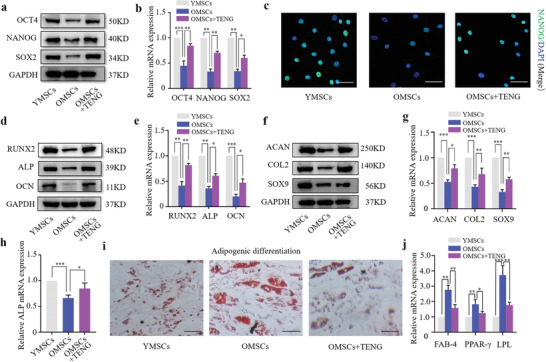
The pluripotency of senescent BMSCs was improved by triboelectric stimulation. a,b) Expression of pluripotency factors assayed by western blot and RT‐qPCR (*n* = 3). c) Immunofluorescence staining of NANOG (green signal) and nuclei (blue signal) analyzed by fluorescence microscopy (scale bar: 50 µm). d,e) Expression of osteogenesis markers assayed by western blot and RT‐qPCR (*n* = 3). f,g) Expression of chondrogenesis markers assayed by western blot and RT‐qPCR (*n* = 3). h) Assay of ALP mRNA expression by RT‐qPCR (*n* = 3). Statistical evaluation was performed by one‐way ANOVA with Dunnett's multiple comparison test (*p < 0.05, **p < 0.01, ***p < 0.001). i) Oil red O staining of lipid accumulation visualized in YMSCs and OMSCs after initiating adipogenic differentiation for 14 d (scale bar: 200 µm). j) Expression of adipogenesis‐related genes (FAB‐4, PPAR‐*γ*, LPL) assayed by RT‐qPCR (*n* = 3).

The regenerative function and differentiation capacity of stem cells greatly decline during aging. The expression of OCT4, SOX2, and NANOG, three transcription factors essential for the maintenance of pluripotency, was significantly increased in stimulated BMSCs (**Figure** **4**a–c and Figure S3a–c, Supporting Information). These results indicated that the pluripotency of senescent BMSCs was enhanced after electrical treatment. Osteogenesis and chondrogenesis of stimulated senescent BMSCs were determined to assess the effects of triboelectric stimulation on differentiation capacity. Senescent BMSCs treated with triboelectric stimulation or not for 7 d were followed by the treatment with osteogenic and chondrogenic differentiation media for 14 d. The data shown in Figure 4d–g indicated that the expression of the markers of osteogenesis (alkaline phosphatase [ALP], OCN, and RUNX2) and chondrogenesis (SOX9, collagen2, and aggrecan) was higher in the group of stimulated BMSCs than that in the control group at the protein and transcript levels. The mRNA expression of ALP was determined using RT‐qPCR and was in agreement with the results of ALP staining. BCIP/NBP, Alizarin Red S staining, and immunofluorescence staining of OPN were used to evaluate the degree of osteogenic differentiation, and ACAN immunohistochemistry (IHC), SOX9 immunofluorescence, and Alcian Blue staining were used to detect the level of chondrocyte differentiation (Figure 4h and Figure S3d, Supporting Information). Furthermore, Oil red O staining of lipid accumulation showed OMSCs stimulated with triboelectric stimulation reduced adipogenic potential compared to OSMCs, which was further demonstrated by RT‐qPCR assay of adipogenesis‐related genes (Figure 4i,j). The results showed a significantly change of expression of these markers after P‐TENG stimulation, indicating that triboelectric stimulation enhanced the differentiation capacity of senescent BMSCs.

**Figure 5:**
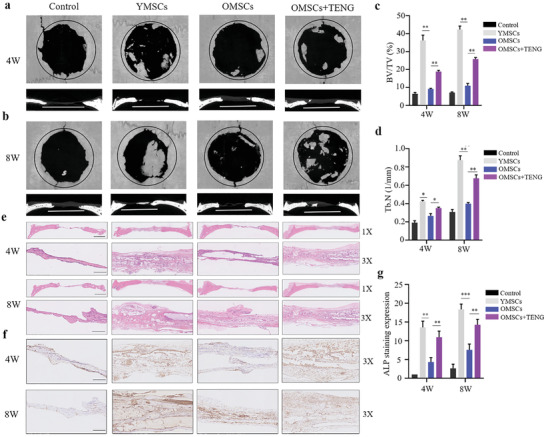
Restoration of the osteogenic potential of senescent BMSCs in vivo by P‐TENG. a,b) Representative micro‐CT coronal and sagittal views of the cranial defect area 4 and 8 weeks after implantation (*n* = 3). The control group was implanted with scaffold alginate gel without BMSCs (scale bar, 5 mm). c,d) Quantification of bone regeneration in calvarial defects. CTAn was used to measure the bone volume density (BV/TV%) and trabecular number (Tb.N, mm^−1^). Statistical evaluation was performed by one‐way ANOVA with Dunnett's multiple comparison test (*p < 0.05, **p < 0.01). e) H&E staining of the cranial defect area 4 and 8 weeks after implantation (Scale bar: 200 µm for 1×; 50 µm for 4×). f) ALP IHC of the cranial defect area 4 and 8 weeks after implantation (Scale bar: 50 µm). g) Statistical evaluation of the ALP staining expression by one‐way ANOVA with Dunnett's multiple comparison test (**p < 0.01, ***p < 0.001).

The osteogenic potential of BMSCs after triboelectric stimulation was detected in vivo to evaluate the rejuvenation effect and specific mechanism of action of TENG on senescent BMSCs. Aged human BMSCs were triboelectrically stimulated for 7 d, and the control groups of senescent and normal BMSCs were maintained under the same conditions in the absence of electrical stimulation. Sodium alginate gel was used as a scaffold of BMSCs for bone regeneration to be implanted in the calvarial defects in rats. Micro‐CT was used to measure new calvarial bone tissue 4 and 8 weeks after the implantation. Micro‐CT images indicated that new bone formation was significantly enhanced in the TENG‐stimulated group compared to that in the control group at 4 and 8 weeks (**Figure** **5**a,b). The BV/TV ratio and Tb.N were used to quantify the growth of new bone; the results indicated that the osteogenic potential in OMSCs stimulated with TENG was higher than that in the control OMSCs group (Figure 5c,d). Moreover, results from H&E staining and ALP IHC further confirmed triboelectric stimulation promoted the OMSCs osteogenesis in vivo (Figure 5e–g). These results support the rejuvenation function of triboelectric stimulation by P‐TENG.

**Figure 6:**
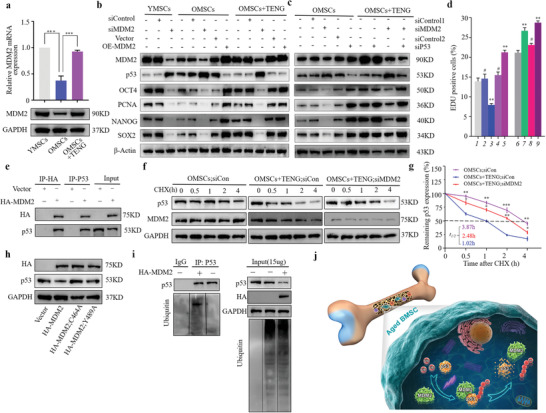
Rejuvenation of senescent BMSCs via MDM2‐p53 pathway by P‐TENG. a) Expression of MDM2 assayed by western blot and RT‐qPCR (*n* = 3). b) Expression of MDM2, p53, OCT4, PCNA, NANOG, and SOX2 assayed by western blot after the transfection with control, siMDM2 or OE‐MDM2 and treatment (*n* = 3). c) Expression of MDM2, p53, OCT4, PCNA, NANOG, and SOX2 assayed by western blot after the transfection with control, siMDM2, or sip53. d) EdU labeling and FACS analysis (*n* = 3) of cellular proliferation (referred to as group 1–9 in Figure S4, Supporting Information). e) Immunoprecipitation analysis of HA‐MDM2‐transfected aged BMSCs followed by western blot with anti‐HA or anti‐p53 antibodies (*n* = 3). f,g) p53 protein stability analysis by western blot after cycloheximide treatment (*n* = 3). h) The expression of p53 assayed by western blot after the transfection of BMSCs with HA‐MDM2 or MDM2 mutants (Y489A and C464A). i) Ubiquitination assay in BMSCs with or without ectopic MDM2 expression. Control IgG and p53 were immunoprecipitated, and western blotting with anti‐p53 and anti‐ubiquitin antibodies was used to detect ubiquitinated proteins (left). Equal loading levels of proteins and global ubiquitination profiles are displayed (right). j) Schematic representation of the mechanisms by which MDM2 regulates p53 after electrical stimulation in aged BMSCs. Statistical evaluation was performed by one‐way ANOVA with Dunnett's multiple comparison test (*p < 0.05, **p < 0.01, ***p < 0.001).

The p53 gene is a critical tumor suppressor, and the overexpression of p53 can inhibit cell growth by inducing cell cycle arrest.^[^ ^18^ ^]^ Senescent BMSCs manifested increased cell cycle arrest and decreased stemness, which was mainly induced by the p53 tumor suppressor. Triboelectric stimulation by TENG reverted p53‐dependent aging of senescent BMSCs. MDM2 is an antagonist of p53 that can destabilize p53 by mediating p53 ubiquitination.^[^ ^19^ ^]^ Activation of the MDM2‐p53 pathway in hematopoietic stem cells can inhibit senescence and rescue oxidative stress‐induced dysfunction of hematopoiesis.^[^ ^20^ ^]^ However, the role of the MDM2‐p53 pathway in P‐TENG‐induced rejuvenation of senescent BMSCs has not been determined. We examined the expression of MDM2 in electricity‐stimulated senescent BMSCs using western blot analysis and RT‐qPCR to determine the function of the MDM2‐p53 pathway (**Figure** **6**a). The results indicated higher expression of MDM2 at the protein and transcript levels, which was consistent with the corresponding changes in p53 expression in senescent BMSCs after triboelectric treatment. Additionally, we silenced MDM2 using siRNA to detect its functional role in the rejuvenation of senescent BMSCs. The results indicated that p53 expression was increased after MDM2 silencing. Moreover, overexpression of MDM2 in senescent BMSCs could inhibit the level of p53, and increased the expression of proliferation and stemness markers. P‐TENG stimulation did not reverse the expression of p53 and other markers of proliferation and pluripotency after MDM2 silencing, indicating the critical role of MDM2 in the rejuvenation process (Figure 6b). Then, siRNA‐p53 was introduced after MDM2 silencing to determine whether the rejuvenation effect was mediated by the MDM2‐p53 pathway. The capacity for proliferation and stemness of BMSCs were subsequently analyzed. Western blot analysis was used to determine the expression of PCNA, SOX2, OCT4, and NAONG (Figure 6c). Consistently, FACS analysis of EdU‐positive cells showed that p53 silencing reversed the phenotype of senescent BMSCs and rescued the effect of MDM2 knockdown on TENG stimulation. This result suggested that triboelectric rejuvenation of senescent BMSCs was mediated by the MDM2‐p53 pathway (Figure 6d and Figure S4a, Supporting Information). MDM2 targets p53 for degradation as an E3 ubiquitin ligase.^[^ ^20^ ^]^ To further investigate the mechanism of interaction of MDM2 and p53 in aged BMSCs, coimmunoprecipitation experiments were carried out in aged BMSCs to pull down ectopically expressed MDM2 or endogenous p53; the results demonstrated a physical interaction between MDM2 and p53 (Figure 6e). Protein stability of p53 was evaluated by cycloheximide pulse‐chase analysis to assess posttranslational regulation of p53. A considerable decrease in the stability of p53 was observed in aged BMSCs treated by electrical stimulation, and this decrease was abrogated by silencing of MDM2 (Figure 6f,g). The E3 ligase function of MDM2 was confirmed to be critical for p53 regulation by introducing MDM2 Y489A and C464A mutants, both of which had no effect on p53 degradation (Figure 6h). Accordingly, ubiquitination analysis revealed that global polyubiquitination was not affected in MDM2‐overexpressing BMSCs, while the level of ubiquitin conjugated to p53 was dramatically increased (Figure 6i). These results indicated that P‐TENG can function as a stimulator of senescent BMSCs, and electrical pulse stimulation can contribute to rejuvenation by activating MDM2‐dependent p53 degradation (Figure 6j). This rejuvenating effect of P‐TENG may be a potential strategy to improve the treatment of patients with osteoporosis, osteoarthritis, or bone defects.

## 3 Conclusion

In summary, we fabricated a P‐TENG that produced stable pulsed current output to stimulate the rejuvenation of aged mesenchymal stromal cells. Compared with direct current which may cause the electrothermal and electrochemical hazards in tissues, pulsed stimulation shows better biological compatibility.^[^ ^21^ ^]^ Our results clearly indicated the antisenescence function of triboelectric stimulation. Senescent BMSCs showed lower SA‐*β*‐galactosidase activity, and the expression of p53, p21, and p16 was significantly declined after 7 d of triboelectric stimulation using electrical pulse. The proliferation of BMSCs was significantly enhanced according to the results of EdU labeling and Ki67 immunofluorescence. The results were consistent with the data of cell cycle analysis. The stemness of BMSCs was increased after triboelectric stimulation, and rejuvenated BMSCs manifested higher osteogenic and chondrogenic potential. And the long‐lasting differentiation period further indicated the triboelectric stimulation could lead to a stable rather than transient pro‐youth effect and nonsenescent BMSCs, which could not be abolished by subsequent in vitro culturing and in vivo transplantation. Loss‐of‐function experiments with MDM2 indicated that MDM2‐dependent p53 degradation was essential for the effects of P‐TENG and was rescued by p53 silencing. Repression of p53 then promotes proliferation and the capacity of differentiation, hence rejuvenating senescent BMSCs.^[^ ^22^ ^]^ These results showed the triboelectric stimulation could function as a stable rejuvenation tactic for senescent BMSCs. However, more studies are needed to explore the other underlying mechanisms and the most appropriate electrical current, stimulation times, and stimulation period for rejuvenation of BMSCs. Furthermore, this study suggests many possibilities for future development of pulsed triboelectric nanogenerators into an implantable device for the treatment of osteoporosis, osteoarthritis, and other diseases associated with aged BMSCs. The study also showed that P‐TENG could be used as an efficient tool for tissue repair and regeneration by in vitro culture of BMSCs.

## 4 Experimental Section

### Experimental Design

In this study, experiments were designed to study the rejuvenation effect of triboelectric stimulation on senescent BMSCs. A P‐TENG that could produce stable triboelectric stimulation was fabricated to stimulate senescent human BMSCs. The behaviors of BMSCs under triboelectric stimulation compared with control group were tested in vitro, including cellular senescence, cellular proliferation, cellular pluripotency, and cellular differentiation. All the BMSCs used in this study were donated from healthy human donors. And in vivo experiments were performed on healthy rats. All the experiments were replicated at least three times.

### Study Approval

Human bone marrow donated by healthy volunteers complied with the protocols by The Ethics Committee of Tongji Medical College, Huazhong University of Science and Technology (No. S347).

All the experiments and procedures on rats were performed in Animal center of Tongji Medical College, Huazhong University of Science and Technology, and all the protocols were approved by The Institutional Animal Care and Use Committee at Tongji Medical College, Huazhong University of Science and Technology (No. S2421).

### Fabrication of P‐TENG

P‐TENG was fabricated using a PMMA sheet with a thickness of 3 mm as the substrate. A square Al foil with a side length of 5 cm and a thickness of 25 µm was used as a triboelectric layer. Two pieces of Cu foils with a thickness of 50 µm were used as two electrodes of P‐TENG. A PTFE film was attached to the Cu foil as another triboelectric layer.

### Cell Isolation and Culture

Human bone marrow specimens were harvested from 12 healthy volunteer donors (for young BMSCs: 3 females and 3 males; age 19.3 ± 1.4 years; for senescent BMSCs: 3 females and 3 males; age 67.6 ± 4.7 years). Isolation of BMSCs was performed using human lymphocyte separation medium (TBD, LTS1077, Tianjin, China) after harvested bone marrow was diluted with PBS (Gibco,14040133, Massachusetts, USA) by 1:1, and then followed by centrifugation at 800*g* for 30 min. After centrifugation, the second layer containing BMSCs was extracted and washed by PBS for twice and then mesenchymal stromal cell medium (Sciencell, No. 7501, California, USA) was used to culture BMSCs with change per 2 d at 37 °C in incubator with 5% CO_2_. Identification of the surface marker of BMSCs was performed by FACS analysis (BD Biosciences, BD FACSCalibur, California, USA) using antibodies shown in Table S2 (Supporting Information). The cells from the first passage were used for following experiments.

### P‐TENG Stimulation of BMSCs

BMSCs cultured in cell culture dish were connected to P‐TENG, and the stimulation in all stimulation experiments was performed for 7 d for 30 min per day. And then the disposed BMSCs were used for following tests or experiments.

### Osteogenic and Chondrogenic Differentiation of BMSCs

Osteogenic and chondrogenic differentiation of all groups of BMSCs, including TENG‐stimulated BMSCs, were separately achieved using human mesenchymal stromal cell osteogenic differentiation basal medium (Cyagen, HUXMA‐90021, California, USA) and human mesenchymal stromal cell chondrogenic differentiation basal medium (Cyagen, HUXMA‐9004, California, USA) for 14 d. Then, following Alizarin Red and BCIP/NBP ALP staining was performed to evaluate the osteogenesis of BMSCs and ACAN IHC and Alcian Blue staining were performed to evaluate the chondrogenesis of BMSCs.

### Alizarin Red S Staining

PBS was used to wash the cells twice followed by fixation using 4% paraformaldehyde for 30 min. Alizarin Red S staining reagent (60504ES25, YEASEN, Shanghai, China) was used for staining for 5 min after washing twice by PBS. Then images were visualized and captured using a microscope (Olympus, BX53; Melville, NY).

### BCIP/NBP ALP Staining

4% paraformaldehyde was used to fix the tissues or cells for 30 min followed by washing by PBS for five times. Then BCIP/NBT Alkaline Phosphatase Color Development Kit (C3206, Beyotime, Shanghai, China) was used to detect activity of ALP according to the instruction for 30 min from light. Then images were visualized and captured using a microscope (Olympus, BX53).

### Alcian Blue Staining

Alcian blue staining reagent (C0153S‐1, Beyotime) was used to stain the slide for 15 min after dewaxing the slides from wax‐embedded tissue using xylene for 10 min. Then solutions at pH 0.2 was used to wash the slides for 30 min. And distilled water was used to wash the slides for three times for 10 s. Then images were visualized and captured using a microscope (Olympus, BX53).

### Immunohistochemistry

For IHC, paraffin‐embedded tissue sections were deparaffinized with xylene and rehydrated with an alcohol gradient and water. Sections were incubated with primary antibodies ACAN (diluted 1:500, ab186414, Abcam) at room temperature for 1 h and biotin‐labeled secondary antibodies for 30 min, and then stained with Vectastain ABC kit and DAB peroxidase substrate kit (AR1000, Boster).

### Animal Model of Bone Defect

All animal experiments were carried out in compliance with a protocol approved by The Institutional Animal Care and Use Committee at Tongji Medical College, Huazhong University of Science and Technology. All experiments were performed on 8‐week‐old male Sprague‐Dawley SD rats. A total of 24 rats were randomly divided into four groups. Models of critical‐sized calvarial defects in rats were generated. In brief, after anesthesia with a 3% (w/v) pentobarbital sodium salt solution (0.1 mL/100 g; Ceva, France), a single 5‐mm defect in the frontal calvarial bone was carefully introduced using a trephine drill. Sodium alginate gel at a concentration of 3% was used as a carrier of human BMSCs isolated and cultured as above protocol, which were stimulated with TENG or not for 7 d firstly. Then, a mixture of the sodium alginate gel and stimulated or unstimulated BMSCs was implanted into the defect of the calvarial bone. The incision was routinely closed with an interrupted 4‐0 silk suture. The sodium alginate gel group were considered as the control groups.

### Micro‐CT Scanning

The new bone tissue of rats was quantitatively analyzed by micro‐CT analysis after sacrifice. Skull specimens were then fixed with 4% paraformaldehyde and scanned by a micro‐CT imaging system (BRUKER, SkyScan 1278, Massachusetts, USA) at a voltage of 50 kV, a current of 145 mA, and a resolution of 9 µm pixel^−1^. CT_VOX_ software was used to visualize the bone structure and CTAn analysis software was used to calculate the trabecular number Tb.N and bone volume/tissue volume (BV/TV) values.

### EdU Labeling

EdU labeling was performed to examine the proliferation status of BMSCs. BMSCs were exposed to 25 × 10^−6^ m of 5‐ethynyl‐2′‐deoxyuridine (EdU, RiboBio, C10338, Guangzhou, China) for 2 h at 37 °C and by fixed in 4% paraformaldehyde. BMSCs were then permeabilized using 0.5% Triton‐X‐100 and then reacted with Apollo488 for 30 min. Subsequently, Hoechst 33342 was used to stain the DNA contents of the cells for 30 min, and images were visualized and captured using a microscope (Olympus, BX53). The experiments were replicated three times.

### RT‐qPCR

Total RNA from the cultured cells was extracted using TRIzol reagent (Invitrogen, 15596026, Massachusetts, USA). The extracted RNA was reverse‐transcribed using a cDNA synthesis kit (Vazyme, NanJing, R312‐01) according to the manufacturer's instructions; cDNA was subjected to RT‐qPCR using RT SuperMix for qPCR (Vazyme, NanJing, R323‐01). The values for telomere length were normalized to the 36B housekeeping control. The primers used for RT‐qPCR are listed in Table S1 (Supporting Information).

### Western Blot Analysis

Western blot was performed after cells were lysed using RIPA buffer (Boster, Wuhan, AR0105), and PVDF membranes were used to transfer the proteins. The membranes were blocked and incubated with primary and secondary antibodies. Then, the images were captured by an image capture system (Bio‐Rad, California, ChemiDoc MP). GAPDH and *β*‐actin were used for normalization. The primary antibodies are listed in Table S2 (Supporting Information). HRP‐conjugated Affinipure goat antirabbit IgG (SA00001‐2, Proteintech, 1:10000) and HRP‐conjugated Affinipure goat antimouse IgG (SA00001‐1, Proteintech, 1:1000) were used as secondary antibodies.

### Cell Cycle Analysis

To measure the cell cycle of BMSCs, cell cycle was first synchronized with serum starvation before the first time of TENG stimulation and then the medium was changed with mesenchymal stem cell medium (Sciencell, No. 7501, California, USA) and started the corresponding stimulation of different groups. During this process, the culture condition between different groups was the same. Then the cell cycle was analyzed at day 7 after the seventh stimulation. Cell cycle analysis was performed using The Cell Cycle and Apoptosis Analysis Kit (C1052; Beyotime, Shanghai, China) according to the manufacturer's instructions. Analysis was then performed using flow cytometry (BD FACS Calibur; BD Biosciences, San Jose, CA).

### SA‐*β*‐galactosidase Staining

Cells were first fixed in 2% formaldehyde and 0.2% glutaraldehyde at room temperature for 5 min and then stained using fresh staining solution at 37 °C for 12 h. SA‐*β*‐gal‐positive cells were counted using flow cytometry (BD FACSCalibur), and images were captured using a microscope (Olympus, BX53).

### Telomerase Enzyme Activity Analysis

The telomerase enzyme activity of BMSCs was analyzed using Telomerase Detection Kit (TRAPeze RT Telomerase Detection Kit; catalog no. S7710; Sigma‐Aldrich, CA, USA), and the experiments were conducted according to the protocol according to the vendor instructions and the final data were acquired by RT‐qPCR.

### Fluorescence Staining of the Cells for *β*‐galactosidase

The cells were incubated with 33 × 10^−3^ m *β*‐galactosidase C_12_FDG substrate (5‐dodecanoylaminofluorescein di‐*β*‐d‐galactopyranoside) in 2 mL medium for 2 h and pretreated with 100 × 10^−9^ m bafilomycin A1 for 1 h at 37 °C. After incubation, the cells were washed with PBS and fixed with 4% paraformaldehyde for 15 min at room temperature. Then, nuclei were costained using 0.1 g mL^−1^ DAPI (Beyotime, Nantong, China). The images were captured using a microscope (Olympus, BX53). The experiments were replicated three times.

### Immunoprecipitation and Western Blot Analyses

Control cells or cells transfected with the expression plasmids were lysed in lysis buffer (1% NP40, and 1% cocktail [Sigma, P8340, St. Louis, MO]) after the corresponding treatments. Lysates were immunoprecipitated (IP) with beads conjugated with anti‐HA or anti‐p53 antibodies (CST). The associated proteins were separated by SDS‐PAGE and probed with anti‐HA or anti‐p53 antibodies.

### In Vivo Ubiquitination Assay

MSCs were transfected with the pcDNA‐HA‐Mdm2 or empty vectors (10 µg) followed by the corresponding treatments. Then, the cells were lysed in cell lysis buffer lacking EDTA and supplemented with 20 × 10^−3^ m imidazole and 10 × 10^−3^ m N‐ethylmaleimide (NEM). Cell lysates (500 µg of protein) were incubated with 20 µL of the beads (QIAGEN) conjugated to a p53 antibody (CST) for 3 h at 4 °C with constant agitation. The beads were washed three times with lysis buffer without NEM. Proteins were separated by SDS‐gel electrophoresis and detected by immunoblotting with anti‐p53 and anti‐ubiquitin monoclonal antibodies.

### Protein Stability Assay

To measure protein stability, MSCs after the corresponding treatments were treated with cycloheximide (CHX, 100 µg mL^−1^) for the indicated times. The expression of p53 was measured relative to MDM2 by western blot analysis.

### Statistical Analysis

Data are presented as the mean ± SD of at least three independent experiments. Statistical analyses were performed using GraphPad Prism 8 software (La Jolla, CA). One‐way analysis of variance (ANOVA) and Student's *t*‐test were used to evaluate the statistical significance of the differences. *P* < 0.05 was considered statistically significant and *P* > 0.05 was considered nonsignificant (ns) (^#^ *P* > 0.05, *P < 0.05, **P < 0.01, ***P < 0.001, and ****P < 0.0001).

## Conflict of Interest

The authors declare no conflict of interest.

## Supporting information

Supporting Information

Supplemental Video 1

## Data Availability

Research data are not shared.
