# Supplementary material for: Rejuvenation of Senescent Bone Marrow Mesenchymal Stromal Cells by Pulsed Triboelectric Stimulation
Source: Adv Sci (Weinh). 2021 Jul 14;8(18):2100964. doi: 10.1002/advs.202100964 (PMC8456218; doi:10.1002/advs.202100964)
Supplement: Supplementary file 1 — Supporting Information [file ADVS-8-2100964-s001.pdf]

WILEY-VCH

© 2021 Wiley-VCH GmbH

ADVANCED SCIENCE
Open Access

Supporting Information

for *Adv. Sci.*, DOI: 10.1002/advs.202100964

Rejuvenation of Senescent Bone Marrow Mesenchymal Stromal Cells by Pulsed Triboelectric Stimulation

Gaocai Li, Qianqian Zhu, Bingjin Wang, Rongjin Luo, Xiaohui Xiao, Yi Zhang, Liang Ma, Xiaobo Feng, Jingang Huang, Xuhui Sun, Zhen Wen, Yue Pan* and Cao Yang**

Supporting Information

Rejuvenation of Senescent Bone Marrow Mesenchymal Stromal Cells by Pulsed Triboelectric Stimulation

Gaocai Li, Qianqian Zhu, Bingjin Wang, Rongjin Luo, Xiaohui Xiao, Yi Zhang, Liang Ma, Xiaobo Feng, Jingang Huang, Xuhui Sun, Zhen Wen, Yue Pan* and Cao Yang**

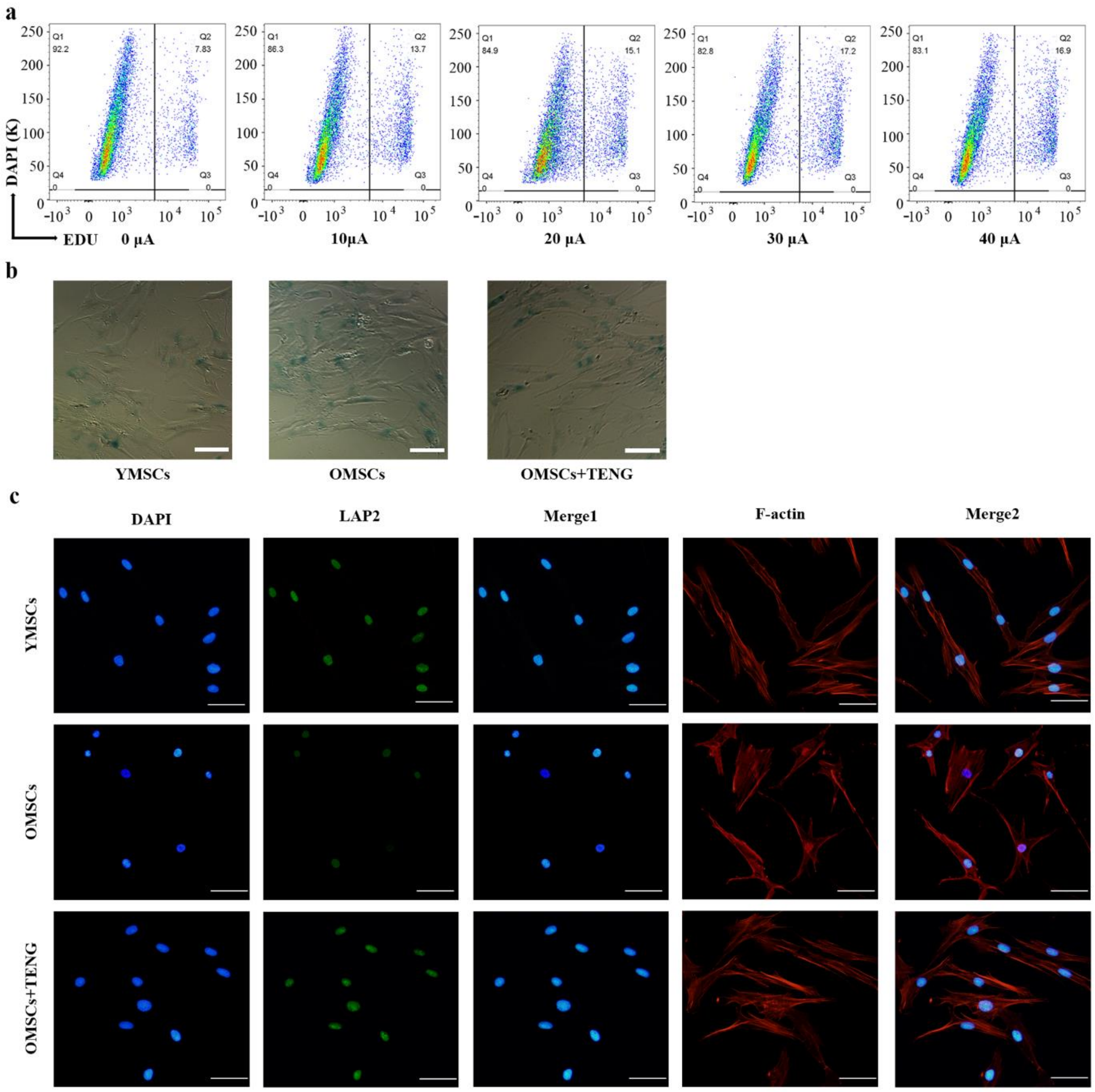

Figure S1. a) EDU labeling and FACS analysis of cellular proliferation under stimulation at different at different current (0, 10, 20, 30, and 40μA, n=3). b) β-galactosidase staining to measure the senescence of the three groups of MSCs (Scale bar: 100μm). c) Immunofluorescence staining of LAP2 (green signal), nucleus (blue signal) and F-actin (red signal) analysis using fluorescence microscopy (scale bar: 20μm).

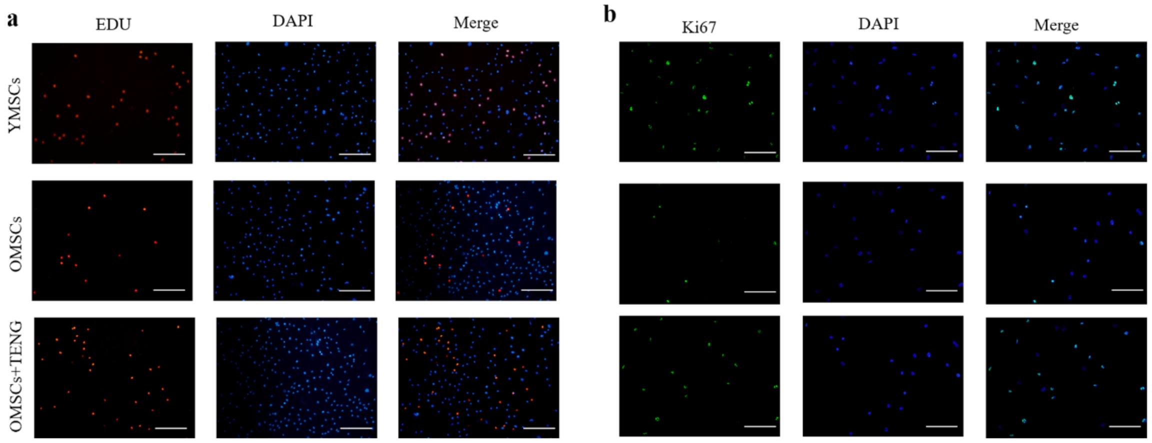

Figure S2. a) Proliferation of the cells by EDU labeling (red signal) and DAPI labeling nucleus (blue signal), (scale bar: 200μm). b)Immunofluorescence staining of Ki67 (green signal) and nucleus (blue signal) using fluorescence microscopy (scale bar: 200μm).

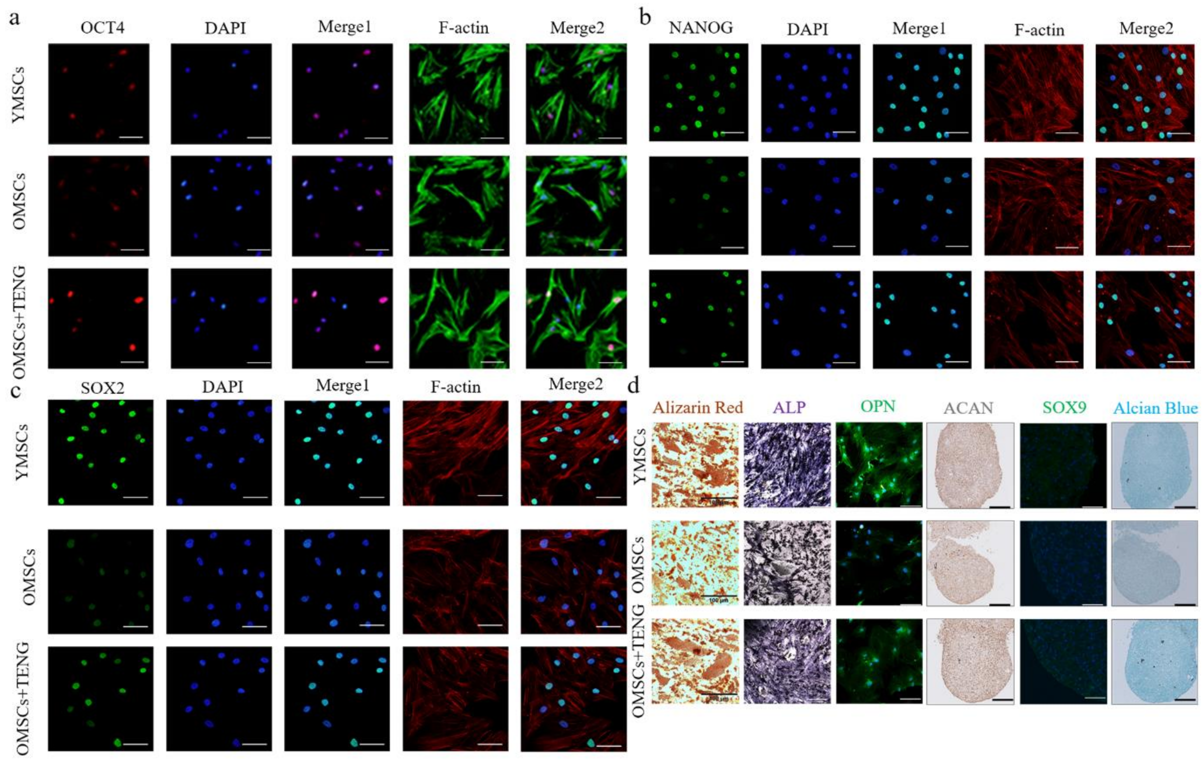

Figure S3. a) Immunofluorescence staining of OCT4 (red signal), nucleus (blue signal) and F-actin (green signal) analysis using fluorescence microscopy (scale bar: 50μm). b) Immunofluorescence staining of NANOG (green signal), nucleus (blue signal) and F-actin (red signal) using fluorescence microscopy (scale bar: 50μm). c) Immunofluorescence staining of SOX2 (green signal), nucleus (blue signal) and F-actin (red signal) analysis (scale bar: 50μm). d) Alizarin Red staining, ALP staining and OPN immunofluorescence (scale bar: 20μm) to evaluate the level of osteogenesis. Immunohistochemistry of ACAN, SOX9 immunofluorescence (scale bar: 50μm) and Alcian Blue staining to evaluate chondrogenesis level (scale bar: 200μm).

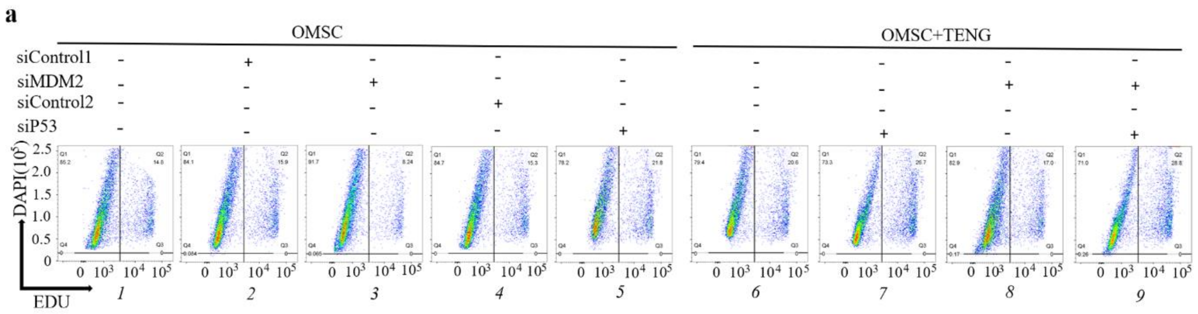

Figure S4. a) EDU labeling and FACS analysis of cellular proliferation under different treatment (referred as group*1*-*9*).

Table. S1. Primers used for RT-qPCR

Homo p53	Forward	5′-TGAAGCTCCCAGAATGCCAG-3′
	Reverse	5′-TGGTGGTACAGTCAGAGCCA-3′
Homo p21	Forward	5′-CTGCCCAAGCTCTACCTTCC-3′
	Reverse	5′- AGGAGAACACGGGATGAGGA-3′
Homo p16	Forward	5′- GACCTGGCTGAGGAGCTG-3′
	Reverse	5′- CGGTAGTGGGGGAAGGCATA-3′
Homo LAP2	Forward	5′- TGAAGAGTGAGTTGGTCGCC-3′
	Reverse	5′- TGCTCTGCCCTTTAGTGGTT-3′
Homo CDC25C	Forward	5′- GTTTGGACCGTGGCCATAGA-3′
	Reverse	5′- TGTTTCCCTGACACGGTTGG-3′
Homo Cyclin A2	Forward	5′- CGTGAAGATGCCCTGGCTTT-3′
	Reverse	5′-AGATGCTCCATTCTCAGAACTTGT-3′
Homo PCNA	Forward	5′-TGAAGCTCCCAGAATGCCAG-3′
	Reverse	5′-TGGTGGTACAGTCAGAGCCA-3′
Homo CDK1	Forward	5′-CCCTCCTGGTCAGTACATGG-3′
	Reverse	5′-TCGAGAGCAAATCCAAGCCA-3′
Homo OCT4	Forward	5′-TCAAAGCAAGCTGGGGAGAG-3′
	Reverse	5′-ACATCCAAGGGATGCAGAGC-3′
Homo NANOG	Forward	5′-CTGCAGAGAAGAGTGTCGCA-3′
	Reverse	5′- ACATTAAGGCCTTCCCCAGC-3′
Homo SOX2	Forward	5′-TTTGTCGGAGACGGAGAAGC-3′
	Reverse	5′-GACTTGACCACCGAACCCAT-3′
Homo RUNX2	Forward	5′- CCGGAATGCCTCTGCTGTTA-3′
	Reverse	5′-ACATCGGTGATGGCAGGAAG-3′
Homo ALP	Forward	5′-AGTGCAGCACGCCTCG-3′
	Reverse	5′-CTCAGTCAGTGCCCGGTAAG-3′
Homo OCN	Forward	5′- TCACACTCCTCGCCCTATTG-3′
	Reverse	5′- AGCCAACTCGTCACAGTCC-3′
Homo MDM2	Forward	5′-AGATGATGAGGACTATTGGAAATGC-3′
	Reverse	5′- TGCACATGTAAAGCAGGCCATAA-3′
Homo Telomere	Forward	5′- ACACTAAGGTTTGGGTTTGGGT TTGGGTTTGGGTTAGTGT-3′
	Reverse	5′- TGTTAGGTATCCCTATCCCTATCCCT ATCCCTATCCCTAACA-3′
Homo 36B	Forward	5′- CAGCAAGTGGGAAGGTGTAATC-3′
	Reverse	5′- CCCATTCTATCATCAACGGGTACAA-3′

Table. S2. Antibodies used in this study

Product name	Catalog No.	Manufacturer	Dilution Ratio
anti-CD73	344015	BioLegend	1:20
anti-CD90	328113	BioLegend	1:20
anti-CD105	323203	BioLegend	1:20
anti-CD34	343603	BioLegend	1:20
anti-HLA-DR	307632	BioLegend	1:20
anti-p53	#2527	CST	1:1000
anti-p21	#2947	CST	1:1000
anti-p16^{INK4A}	#80772	CST	1:1000
anti-CDC25C	ab32050	Abcam	1:1000
anti-Cyclin A2	ab181591	Abcam	1:1000
anti-PCNA	#13110	CST	1:1000
anti-CDK1	19532-1-AP	Proteintech	1:500
anti-OCT4	ab19857	Abcam	1:1000
anti-NANOG	ab109250	Abcam	1:1000
anti-SOX2	#3579	CST	1:1000
anti-RUNX2	#12556	CST	1:1000
anti-ALP	DF12525	Affinity	1:500
anti-OCN	DF12303	Affinity,	1:1000
anti-ACAN	ab186414	Abcam	1:1000
anti-COL2	ab188570	Abcam	1:1000
anti-SOX9	#82630	CST	1:1000
anti-MDM2	ab226939	Abcam	1:1000
anti-GAPDH	BM1623	Boster	1: 20000
anti-Ubiquitin	#3936	CST	1:1000
